# Public responses to volunteer community care: Propositions for old age and end of life

**DOI:** 10.1371/journal.pone.0218597

**Published:** 2019-07-01

**Authors:** Alan Tapp, Clive Nancarrow, Yvette Morey, Stella Warren, Nicola Bowtell, Julia Verne

**Affiliations:** 1 Faculty of Business and Law, University of the West of England, Bristol, United Kingdom; 2 National End of Life Care Intelligence Network, Public Health England, Bristol, United Kingdom; University of Auckland, NEW ZEALAND

## Abstract

**Background:**

Funding shortages and an ageing population have increased pressures on state or insurance funded end of life care for older people. Across the world, policy debate has arisen about the potential role volunteers can play, working alongside health and social care professionals in the community to support and care for the ageing and dying.

**Aims:**

The authors examined self-reported levels of care for the elderly by the public in England, and public opinions of community volunteering concepts to care for the elderly at the end of life. In particular, claimed willingness to help and to be helped by local people was surveyed.

**Methods:**

A sample of 3,590 adults in England aged 45 or more from an online access panel responded to a questionnaire in late 2017. The survey data was weighted to be representative of the population within this age band. Key literature and formative qualitative research informed the design of the survey questionnaire, which was further refined after piloting.

**Results:**

Preferences for different models of community volunteering were elicited. There was a preference for ‘formal’ models with increased wariness of ‘informal’ features. Whilst 32% of adults said they ‘might join’ depending on whom the group helped, unsurprisingly more personal and demanding types of help significantly reduced the claimed willingness to help. Finally, willingness to help (or be helped) by local community carers or volunteers was regarded as less attractive than care being provided by personal family, close friends or indeed health and care professionals.

**Conclusion:**

Findings suggest that if community volunteering to care for elderly people at the end of life in England is to expand it may require considerable attention to the model including training for volunteers and protections for patients and volunteers as well as public education and promotion. Currently, in England, there is a clear preference for non-medical care to be delivered by close family or social care professionals, with volunteer community care regarded only as a back-up option.

## Introduction

Funding shortages, demographic and cultural changes have increased pressures on state or health insurance funded health and social care for the elderly including those approaching the end of life, and contributed to a more general reflection of how best to care for older people in many (particularly 'Westernised') countries across the world [1–5]. The populations of industrialised countries are ageing rapidly, for example in England two thirds of people who die are now aged 75 and older, and 38% aged 85 and older, with projections showing increased numbers of elderly people needing end of life care [6–8]. In addition, death in old age is often preceded by a period of slow decline and increasing frailty and dependence on others for care. Inadequate community care provision can lead to unwarranted emergency admissions [9] and “bed blocking” in which elderly people are kept in hospital simply because no suitable care option can be found at home or in a care institution [10]. Government policy makers across the World [11] have therefore considered how to meet the needs of elderly people at the end of life by supplementing professional services (in particular the National Health Service and Social Care Services) with care provided by families and/or volunteers [12]. The current national end of life care strategy for England described in the Ambitions Framework dedicates a section to the role the community can play in supporting patients and families at the end of life [13]. The recently published NHS 10 year Plan for England also identifies a need for more care, including end of life care, to be delivered in the community and highlights the role of both families and volunteers in supporting professionals [14].

This approach may also be popular as elderly people generally prefer to stay in their own homes as long as possible and try to delay being admitted to institutions such as care homes [15–18]. Families have been recognised as important providers of day-to-day care for the elderly, supported by professionals (medical and social care input) [19]. However, there are many elderly people who have no family or whose family cannot provide all the care needed [20].

Volunteers have played a pivotal role in the delivery of palliative and end of life care since the foundations of hospices in the 1960s. Indeed Dame Cicely Saunders, the founder of palliative care and the modern hospice movement greatly valued the role of volunteers on several counts [21]. Firstly, she wanted the volunteers to represent the patient and family population using the hospice in the belief that this would offer comfort and familiarity to the patients and families. In effect, she wanted the hospice in-patient unit to still feel part of the community from which the patients came. Dame Cicely also understood that the volunteers would act as ‘educators’ for the local community from which they came and help to change knowledge and attitudes about the role of hospices. Thus in England the role of palliative care volunteers has been closely linked to hospices, largely working in the inpatient units [22] but more recently out in the community as in, for example the Compassionate Neighbours Project [23]. In other countries, for example Austria, The Netherlands and in the North of Poland around Gdansk, the palliative volunteer movements started in the community [24–26]. Rather than being driven by a medical model of caring, these movements had more of a social solidarity origin. Unlike in England, volunteers in end of life care tend to undertake personal caring duties such as washing and feeding for the patients in their homes as well as providing companionship and less demanding tasks such as shopping. Similar models promoting the concept that ‘care is everyone’s business’, have been inspired by community care norms elsewhere, in for example India [27], and these in turn have influenced the UK’s Compassionate Communities and Compassionate Cities movements [28–29].

In re-building community centred care, 'Compassionate Community' proponents advocate partnerships between communities and professionals, but with a reversal of the locus of control away from medical professionals making decisions to key decisions instead being made by the community themselves, with the expected norm being that old age and end of life is spent at home unless exceptional circumstances intervene. An increasing literature now exists to describe and explain these 'Compassionate Community' approaches [30]. A core ethos is that care rests on the development of empathy in a community created through shared narratives of concern and a duty of care [31], with a paradigm shift from clinical ethics to community ethics. A second principle is that community care rests upon the assets contained within the community and these are energised through 'asset based community development' [32] approaches, supported by clinical experts.

However, a recent critique [33] has highlighted the difficulty of deliberate, structural attempts to create compassion within modern individualistic societies: as Zaman et al. put it, ‘[there are] difficulties for compassion to flow freely, particularly within Western society’ (p141).

There have been a number of initiatives described in the literature on aged and end of life care covering practical issues of training and examining the impact of volunteering on the recipients and providers [34–35]. This literature is quite eclectic, covering different dimensions of the practice. Hospice based volunteering has been supplemented by an increase in activity by charitable organisations representing older adults to promote volunteering to help people in their homes, particularly to combat loneliness [36–37]. Further afield, there is an excellent international example of state supported campaign for mass volunteering in end of life care in Kerala [38]. However, as we have discussed, Zaman et al. point out it is not clear whether the practices from Kerala [39] would be transferable to other contexts such as, in the case here, England. In England, apart from a few local areas, the concept of volunteer provision of end of life care in peoples’ homes alongside professionals has not been widely explored or implemented One of the consequences of the shift to institutional care for the elderly in England has been the widespread de-skilling of communities in relation to dying, bereavement and care-giving [40]. Perhaps there may be lessons drawn from other fields about mobilising volunteers, for example to support the London Olympics [41]. The National Health Service in England is actively promoting the concept of volunteers [42]not least with the support of social marketing approaches such as those used by the *Dying Matters* organisation [43]. However, as Scott and Howlett noted [44], although there are plenty of descriptions of models and experiences of the role of volunteers in palliative and end of life care, there has been no research testing the views of the general public in England towards volunteering in the community to help the elderly at the end of life alongside health professionals. In particular, there is as yet no published assessment of the extent to which people generally would be prepared to help strangers as opposed to family (or people that they know), how many hours they would be prepared to give, what type of care they would be prepared to undertake as volunteer carers, and what support and/or protections they feel they would need.

Understanding public opinion in these regards is important in informing effective policy. In response, a representative survey of adults (aged over 45) in England was undertaken. In this paper the authors report on claimed levels of care *already* provided for the elderly (mainly by close family), reactions to various considerations in volunteering, claimed willingness to help others locally, and disposition to receiving help from local people. The primary research objectives were:

- to establish reported current levels of informal voluntary care for old age and end of life i.e. the percentage of the population of England aged over 45 that have provided care, not as part of their employment, and the types of care provided;
- to explore public attitudes to the concepts of local groups helping with old age and end of life;
- to estimate preferences of how such groups may be designed (formal, i.e. with professional structures and help as a dominant feature, versus informal, i.e., organised and run by the community themselves); and
- to explore levels of public willingness to help and willingness to be helped.

## Method

Questionnaire design and data interpretation were undertaken by the authors. Online survey data collection, data processing and tabulations were sub-contracted to a well-known and reputable independent research provider, YouGov–a Member of the British Polling Council. The study focused on those aged 45 and older (hereafter 45+), given the younger members of this age group represent those who may be the first to be called on to help older relatives, and who may also envisage requiring help in later years. The sample also included the elderly who might already or soon need assistance or have experience providing it to spouses or others.

The design of the online questionnaire and in particular the generation of response options for questions were informed by three stages of formative work: first, key literature [45–47] on volunteering for the elderly, including an internal report on volunteering (see link in Supporting Material); second, a stage of exploratory qualitative research consisting of adults aged 45+ comprising twenty depth interviews, four group discussions, and sixty semi-structured interviews [48], and finally, a pilot survey of the questionnaire design. The pilot comprised 201 online access panellists (all aged 45+ but split by 53% aged 45–69; 47% aged 70+). Importantly the pilot sought respondents’ comments on the questions and the response options to ensure all salient issues were covered in the final questionnaire. The final questionnaire used only closed pre-coded response options, including where appropriate an “others” option.

These three stages of preparation for the main survey helped in the format of questions and response options for:

1. Levels of care for the elderly. Who was helped and in what ways.
2. General opinion on a community care proposition presented.
3. Features of care groups that might/might not appeal to the public.
4. Willingness to join a care group and types of care that could be provided.
5. Willingness to receive community care.

YouGov recruited a total sample of 3,590 adults aged 45+ from their online access panel between September 27 and November 7 2017. Demographic quotas were set using the UK's Office for National Statistics (ONS) projections and data for England as of September 2017 to reflect the population. Sampling was random within quota cells (a stratified random sample). To ensure statistically reliable bases for older respondents a boosted target sample size of 1,000 was set for those aged 70–79 and a boosted sample size of 500 for those aged 80+. The survey data was then weighted to be representative of the overall population aged 45+ residing in England using ONS projections and data.

Face saving question formats were used to reduce social desirability bias and, where appropriate, randomisation or rotation were used to reduce order effects. Administration online allowed respondents to work though the questionnaire at their own pace, deemed particularly important for some older respondents, enabling more considered opinions [49–51]. A link for the full questionnaire can be found in the Supporting Materials section of this paper.

YouGov achieved a 90% completion rate (90% of those starting the online questionnaire completing it) yielding an overall 48% response rate among those invited. Importantly the subject was not off-putting given the topic was only revealed once respondents opened the survey, as 90% then completed it. YouGov incentivise their panel members to participate in surveys with a “points for gifts” scheme. Further, key demographics were representative of the population as a result of quotas set and weighting. Tabulations were provided by YouGov using their proprietary software (Gryphon). Tabulations were based on the sample of 3,590 with no individual respondents being identified nor identifiable. YouGov, as a Member of the British Polling Council, also safeguard the identities of respondents [52].

### Ethical approval

The research was approved by the University’s Health and Applied Sciences Faculty Ethics Committee UWE REC REF No: HAS.16.12.068.

## Results

### A note on the sampling error associated with table percentages

To help judge the likely statistical reliability of the percentages in the paper there is a table in Supporting Materials which shows the margins of error for the base sizes and % results used in all Tables at the 95% confidence interval.

### Levels of local care provided

We began by ascertaining reported levels of care already being undertaken, now, or in the past, by the sample of respondents. Sixty one percent of respondents (aged 45 or older) claim to have (ever) given help to an elderly person needing care for old age/end of life, with 23% claiming to have done so in the last year.

However, propensity to help non-family (neighbours, friends, and even local people not known to the helper) is key to a community model. Accordingly, those who have cared for someone elderly were asked who they cared for and the types of help provided.

Elderly relatives were the group most often mentioned as being helped (92% of mentions), while 17% helped a close friend or neighbour. Only four percent helped someone they were acquainted with but did not feel close to and only 3% (Not significant vs 4% fig at 95% CI) helped someone they did not know at all at first (Table 1).

**Table 1:** Recipients of care.

Base: Adults aged 45+ who have cared for an elderly person	n = 2219
Who received care?	
A relative/a family member not living with you **	65%
A close friend/neighbour	17%
Another family member living with you (not husband or wife)**	15%
Husband/wife/partner**	12%
Someone you were acquainted with but did not feel close to	4%
Someone you did not know at all at first	3%
Other	2%
Total mentions of family members	**92% **

Note: All ** in Table 1 are family members.

Note: The percentages column totals 122% as some respondents had helped more than one person.

Table 2 also reports the types of help given in descending order. Not surprisingly, fairly simple tasks (checking if OK, companionship, shopping) were most often claimed, whilst personal or medical care were much less prevalent.

**Table 2:** Type of care given.

Base: Adults aged 45+ cared for an elderly person	n = 2219
Types of care given	
Checked if they were OK/ kept an eye on them	72%
Conversation/companionship	72%
Shopping	70%
Household chores (cleaning, making the bed, etc.)	53%
Provided transport	52%
Prepared meals	49%
Collection of prescriptions and/or organising tablets for the day/week	45%
Washing and drying clothes	42%
Managing correspondence	38%
Personal care and hygiene (help in the bathroom, toilet)	29%
Gardening	29%
Lifting them/assisting to move	28%
Financial advice	25%
Pets (care, exercise)	8%
Injections for, say, pain relief	4%
Other types of help	18%
None of these	1%

### General opinions on community help

This vision of community care was presented to respondents:

*There is a view that there may not be enough health and social services to fully look after everyone in their old age. One idea is to encourage local people to help those who are elderly and need care for problems related to old age. They may also help the elderly who need care at the end of their life*.

*Those providing help would be supported by the NHS and Social Services*.

*Importantly, with such help it could mean people have the choice of staying in their homes longer rather than going into care and nursing homes or hospitals*.

Respondents were presented with a number of statements about this vision and were asked how strongly they agreed or disagreed with each one. Table 3 examines the levels of agreement with various opinions about community help. A clear majority see the merit of the idea (first three rows of Table 3) but there is some possible resentment too (a sentiment that the state should provide).

**Table 3:** Opinions on or related to the community care concept.

Base: Adults aged 45+ (n = 3590)	Total Agree	Neither Agree nor Disagree	Total Disagree
It will increasingly be needed	80%	16%	3%
It could help many of the elderly in need	78%	17%	5%
This is a good idea	69%	19%	11%
It shouldn’t be necessary, the state should provide	51%	30%	19%
Their own family should provide this care	48%	37%	15%

Note: Row % adds up to 100% (though rounding may show as 99% or 101%)

There is, perhaps, evidence that these opinions are lightly held and prone to change—shown by a high percentage of respondents agreeing with contradictory statements. For instance, 32% of the total sample agreed with *both* “*It is a good idea*” and “*It shouldn’t be necessary*, *the state should provide*”. Similarly thirty-six percent of the total sample agreed with both "*It is a good idea*” and “*Their own family should provide this care”*.

### Designing care groups that appeal to the public

In order to test preferences for informal, community controlled volunteering or more formal authority-led volunteering, the relative appeal of formal features (identified in Table 4 by italics) versus informal features was ascertained. As can be seen, there is generally a preference for formal designs with increased wariness of informal features such as groups making decisions on their own, or groups not bound by outside rules. Groups designed around fixed levels of commitment, and designs that helped strangers as opposed to people known also reduced appeal. Many features that appealed had cost and resource implications: the more formal model components implied the desire for health and social services support, training, insurance, police checks, codes of conduct, and possibly confidentiality agreements.

**Table 4:** Appeal of features of a local group helping the elderly.

FORMAL FEATURES in italics Base: adults aged 45+ n = 3590	Total for whom it appeals	Total for whom it is off-putting
*Some local groups are formal*, *co-ordinated and organised with the NHS/Social Services closely involved *	65%	6%
Some local groups have set up on their own and are informal, unofficial and not bound by outside rules or interference but can call on help from the NHS or Social Services	46%	20%
**HOW GROUPS MIGHT WORK **		
In some groups district nurses and social services support helpers with advice	85%	2%
*In some groups members would have the chance to undertake any training they thought would benefit them *	81%	3%
*Some groups include police checks when they screen new members *	77%	5%
The team members cover each other to make sure there are no gaps in the aid offered to a person	75%	3%
*Some groups take out insurance in case of accidents *	74%	5%
*In some groups members would be asked to sign a code of conduct—a set of rules and guidelines on issues such as confidentiality *	73%	6%
*Some groups have a formal process for interviewing and screening possible new members to join the group *	67%	9%
*In some groups*, *group members would be expected to follow guidelines set by experts such as the NHS and Social Services *	64%	8%
Some local groups make decisions on their own as to who does what and when	39%	20%
**TIME DEDICATED **		
In some groups members can contribute on an occasional basis, when they can	67%	6%
In some groups members are generally expected to commit to a fairly fixed timetable	32%	29%
**RECIPIENTS OF HELP **		
Groups might help people you know well (relatives, friends, neighbours)	63%	5%
Groups might help people you don’t know at first	25%	21%

Note: responses for ‘neither appeals nor puts off’ were not shown for brevity. If these were included each row adds up to 100%.

### Willingness to join a local care group

Personal willingness to help was assessed by asking respondents whether they might join a group with features that *appealed to them personally* (Table 5). Under these conditions, thirty-two percent said they *might* join depending on whom the group helps, the time involved, and how it is organised.

**Table 5:** Disposition to joining a local group helping the elderly.

Adults aged 45+: Unweighted base	n = 3590
Disposition to join a local group	
I might join depending on the nature of any involvement (who the group helps, the time involved, how it is organised)	32%
My health or age would rule me out of joining such a group	25%
My other commitments would rule me out of joining such a group	15%
I already belong to a care group for the elderly	1%
I would prefer to provide care on my own	11%
For other reasons I wouldn't be interested in joining such a group	16%

Note: Only one response permitted (totals 100%)

Table 6 also indicates the type of help that the 32% of helpers might provide. Responses are presented in descending order of frequency of mention. Unsurprisingly the more personal and demanding types of help are mentioned less often.

**Table 6:** Type of help offered.

Base: Would provide care for:	Someone close	Someone not known
Types of care would provide	n = 1050	n = 785
Check if they are OK/ keep an eye on them	84%	80%
Shopping	79%	75%
Collection of prescriptions and/or organising tablets for the day/week	74%	71%
Conversation/companionship	76%	75%
Prepare meals	51%	47%
Provide transport	52%*	44%
Pets (care, exercise)	50%	47%
Managing correspondence	50%*	43%
Household chores (cleaning, making the bed, etc.)	47%	44%
Washing and drying clothes	44%	41%
Gardening	43%*	36%
Financial advice	28%*	22%
Injections for, say, pain relief (assuming trained/qualified)	16%	14%
Personal care and hygiene (help in the bathroom, toilet)	14%	12%
Other types of help	1%	1%
None of these	1%	2%
Not relevant	2%	3%

*significant difference to column opposite at 95% CI

Note: Respondents can give more than one answer

Given the possible policy interest in a more ‘radical’ option of creating a new norm for general community wide help for older people, the survey included a question on willingness to help elderly people *not known* to the respondent. Here, only twenty two percent said they might join a local care group with the features they liked with the remainder either not being in a position to help (53%), or expecting their family (18%) or friends/neighbours (7%) to look after them, reinforcing the observation that emotional and physical distance are likely barriers to helping elderly strangers.

If community care models are to be a success then strong promotion is likely to be important. Hence, respondents who indicated they might join a local help group for the elderly were asked why they might be interested in joining. “Feel good” psychological boosts were key factors with a net cumulative of 82% signalling these as reasons. These included ‘feeling good helping’ (48%), ‘spending my time more meaningfully’ (46%), ‘meeting other people’ (35%), and ‘providing a new interest in life’ (22%). However, self-interest was also a key factor with sixty one percent agreeing ‘I might need help myself one day from the group’ and 27% agreeing ‘I know people who will need care’. Altruism also featured with agreement that ‘helping others is morally the 'right' thing to do’ seen as a reason to join by 58%.

Finally, personal characteristics data (socioeconomics, age, health and marital status) were gathered and examined in order to compare respondents who said they ‘might join’ a local care group versus those who said they would not join such a group. In general, younger and wealthier respondents in good health and living in more prosperous locations were more likely to help.

### Willingness to receive community help

Respondents were asked (Table 7) *if you were judged at some point in the future as needing care for age related problems or were close to end of life*, *which of these would be your first choice for care*? *And then your second*, *third and fourth choices*? As can be seen [highlighted in bold], local community carers or volunteers were regarded as less attractive than personal family, close friends or health and care professionals–perhaps illustrating how known options tend to be preferred over the unknown.

**Table 7:** Preference for type of care should the need arise.

Adults aged 45+ (n = 3590)	1^st^ choice	1^st^ 2^nd^ or 3^rd^choice
Your family/a family member caring for you in either your home or their home supported by health professionals	48%	72%
Visiting carers provided by NHS/Social Services	17%	62%
A care/nursing home	13%	44%
Close friends caring for you in your home supported by health professionals	9%	44%
A hospital	5%	25%
**Local people you know caring for you informally in your home supported by health professionals **	5%	30%
**A group of volunteers you may not know caring for you in your home supported by health professionals **	4%	22%

## Discussion

As the population ages and health and social care services are under greater pressure to provide care for people ageing or at end of life, new models of care which involve more input from families and volunteers may need to be developed. While the role of volunteers caring for the elderly in the community is well established in many countries across the world, it is not yet common practice in England. To inform policy debates a representative survey of adults aged 45+ in England was used to explore public preferences for the type of care they would be willing to provide or receive.

More specifically, we sought answers to four questions:

- to establish reported current levels of voluntary care for old age and end of life;
- to explore public attitudes to the concepts of local groups helping with old age and end of life;
- to estimate preferences of how such groups may be designed; and
- to explore levels of public willingness to help and to be helped.

More than half the sample surveyed claimed to have (ever) given help to an elderly person needing care for old age/end of life, with nearly a quarter claiming to have done so in the last year. However, the majority of the care provided was in a light form, as opposed to more demanding, and given to family. This response may be regarded positively or negatively depending on perspective: on the one hand, a high proportion of older adults therefore have experience of care, however, on the other hand the tendency to assist with light care may indicate a reluctance to take on additional care. Moreover, the proportion of people helping someone they were only acquainted with or did not know at all was relatively low. Therefore, the idea of community carers helping local people they do not know, if it is to grow, would have to start from a low base. That said, in exploring public attitudes we found majority support for the basic idea of community care while also noting some resentment that the state should provide.

In exploring preferences for different designs of local care groups we found a clear preference for 'organised' descriptors over 'informal' or 'unofficial' designs for the model of volunteering. Indeed, there was general wariness of features such as groups making decisions on their own, or groups not bound by outside rules. Perhaps most crucially, individual willingness to help and to be helped were examined. Whilst the 'headline' figure of 32% of adults aged 45+ indicating willingness to help seems good news, this is mitigated by the reluctance to help with more important, difficult or committing tasks such as 'providing personal care and hygiene' compared to easier and less committing care such as 'checking if someone is OK'. Not only this, but in assessing the translation of the thirty-two percent survey response to actual behaviour, we note that, in reality, it is unlikely one could re-create the blanket awareness, invitation to join or personalised design features of a help-group that the authors artificially created in the survey setting. Finally, in surveying preferences for *being helped*, unsurprisingly perhaps, people very strongly preferred 'family' over local community or volunteer help.

These findings have implications for public policy. They suggest that if wide ranging community-controlled care is to succeed it would require considerable public education and promotion. The primary preference was for care delivered by close family or by health professionals, with community care of any type regarded only as a back-up option. There was some willingness to join a structured group of volunteers. However even within this format it was striking that willingness to help dropped steeply when directed at strangers or undertaking more demanding tasks normally carried out by professional carers. In addition, there was a reluctance to commit to structured timetables which suggests a willingness to only commit when it suits.

The downbeat public response reported here should be accompanied by caveats. The preferences expressed for existing (known) sources of support (NHS, Social Care) over an untried, unknown initiative are understandable. Whilst the crises in public funding of old age and end of life care have been very newsworthy across many countries internationally as well as in England, locally the focus of this news has been largely confined to the lack of money to pay for existing institutional care or social care at home [53]. There has been little coverage of alternative options such as those debated in this work, and so there is little public recognition of community alternatives, and little time for such ideas to gather momentum. In particular, the 'utopian vision' depicted by the Compassionate Communities movement that care is everyone's duty is not yet evident in general, mainstream public discourse [54–56]. The results presented here suggest that their vision—to turn *personal* choices of whether to help or not into *social expectations*, obligations and even norms where locals rally round to help each other as a matter of everyday habit–will require considerable promotional work and policy support if it is to succeed in persuading the public to rally to their cause and may be associated with ethical challenges [57–58]. There are examples of successful promotional work internationally: in Poland for example, great effort has been put into raising public awareness to promote hospice volunteering. A successful national campaign ran between 2007–10 by the Hospice Foundation called ‘I like helping’ [59]. That said, in assessing international comparisons of hospice volunteering it should be noted that in different countries there may be a greater desire to assist in an institutional context rather than within communities.

In seeking reasons for the apparent reluctance of English respondents to commit to more radical options the authors noted the cultural doubts about the applicability of such a model to 'westernised' countries. In particular as noted earlier Zaman and co-authors contend that there are difficulties for compassion to 'flow freely', particularly within western society. This may be because of what they term specific socio-political structural factors such as the atomisation of modern westernised life, the individualism that dominates modern living and the importance placed on privacy and personal space therein (p140). They raise concerns about the difficulty of breaking down highly professionalised health structures, not least because of anxiety about litigation on health and safety grounds, but also a 'context of suspicion and mistrust within the global political scenario' [60] (p140). They also point out that compassion is not merely a passive sense of pity, it is also about engagement—seeking to assist those whose suffering may be ameliorated by our actions. However, compassion is not the only issue, and comparison of the evolution of models of volunteering in palliative care by Scott and Howlett [61], especially in the community, elucidates the role of history, politics, religion and traditions as well as government policy in the level and type of volunteering seen.

It is questionable whether all these cultural barriers can be overcome, but if so new policies are required to support community care. The mix of activities required might include [62]: comprehensive and long-term education, advocacy, community development, and service provision re-directed to communities. Asset-based community development (ABCD) approaches to health and well-being are distinctive because they focus on identifying and building on the strengths or “assets” of individuals and communities [63]. ABCD is best used to highlight positive capability, changes in attitudes and values, personal and collective empowerment, and raising the self-esteem and resourcefulness of individuals to improve and sustain their own health. In theory therefore ABCD seems an appropriate way to create the culture change required to make community care a success. Indeed, locating control of care within the community itself is expanded upon in the Indian Kerala case study that has been reproduced in a European setting in Switzerland [64], with community based palliative care forums in which local representatives play a decision making and organising role for community end of life care, supported by professional medics. We noted above how similar models which evolved independently can be seen in the Netherlands and Poland, and they are also being actively promoted in Latin America as a way to deliver palliative care to all [62–66]. Others however contend that currently there is a limited evidence base for the effectiveness of ABCD in supporting people with long-term health problems [67–68]and, therefore, expanding the scope of ABCD supported community care could be a challenge.

Other (arguably more direct) approaches are available to change behaviours, of which one such is the use of social marketing. Social marketing is often regarded and deployed as a set of communications tools, but a more comprehensive view involves the use of commercial marketing principles in a health or social setting [69]. Initially, social media and public relations campaigns could be used to strengthen the rather superficial level of attitudinal support found in this work. It is recognised that community-centred care initiatives may meet with media and other organisational opposition ('the state should be providing'), however, this data also highlights that, while not underestimating the difficulties, beliefs may be strengthened on this subject. The need for professional public relations campaigns is apparent.

More substantially, marketing offers the potential for 'exchanges' offered to the public, for example a social contract in which people receive some form of exchange or payment in return for care, something already undertaken in a limited way in England with the use of social care payments. It has been shown that although volunteers are not necessarily motivated by incentives, they sometimes recognise and appreciate rewards [70] helped by matching recruitment messages to the motivational needs of volunteers [71]. These ideas could be operationalised by using personal health budgets in England [72]such that social care resources currently used to pay for hospital care could be controlled by individuals who 'pay' neighbours to look after them. However, there is little doubt that promoting rewards and exchanges may jar with advocates of the ethos of community care being a privilege rather than a burden, and care being a natural part of community life [73]. Therefore, it may be that social marketing principles may be better used to promote and operationalise a model of volunteering which—according to our results—meets with more public acceptability.

The Zaman et al. critique of compassion and community care may be a reminder that investing in these areas is not without risk. Thus, even if the above measures were to be fully resourced, it is possible that the cultural climate of England is simply not ready for 'care is everyone's business'. Perhaps English people are just too private, too individualistic [74], too cautious about interfering in others’ business, or too set in their ways to take up the compassionate community ethos apart from in community pockets such as small middle class rural hamlets. Indeed, on this specific point we noted earlier that our data suggests those more willing to help are somewhat younger, wealthier and from more prosperous areas than average. This may reflect the longstanding tradition in England of the noblesse oblige ‘duty to the poor’ model which has had such a strong impact on the culture of volunteering [75].

In understanding these variations between different demographics and indeed between national dispositional differences, Zaman et al.'s question of 'intentional creation of compassion' may need deeper study in the context of different countries. Such research may yield vital insights that would provide the platform for national decision making on whether allocating public resources to community-based care would be effective. It is interesting that the biggest success of community care of older people internationally appears to reside in India, a country with quite a strong collectivist culture—in opposition to the individualistic cultural norms of England. Internationally, community volunteering for old age and end of life care seems to flourish in societies with a more collectivist approach, where volunteering is seen as social solidarity with the elderly and dying. Indeed, in Germany the term for volunteer is ‘Ehrenamt’ (honorary office) and the political term ‘Bürgershaftliches Engagement’ (civic engagement) bestows the concept of a noble function for society on the role of volunteering [76].

## Strengths and limitations of this research

This survey focused on the 45+ age group as the younger end of this spectrum is likely to have older relatives that might need care. The older end of the spectrum, of course, might also provide care as well as might need care in the future. It was judged that care for the elderly would be likely to come from middle-aged family members rather than the older age group largely for health related reasons such as reduced mobility, strength, cognitive ability and ability to drive. This was supported by the data. Of course, those aged under 45 might also volunteer to care for the elderly but might be less able to do so given career, familial and financial pressures. Their focus for volunteering might be in other areas given their middle-aged parents are likely to be seen to be the first to be called on. This seems a reasonable assumption but it is recognised that confining our survey to 45+ adults is a potential limitation as some under 45’s might volunteer but it was judged to concentrate our resource on the 45+ age group being the more likely.

All modes of survey data collection (telephone, face to face, postal, online) have their potential biases (sampling bias in terms of coverage, non-response bias, socially desirable responding bias, and so on). The key strengths of this online research include the attempts to reduce bias from socially desirable responding by interviewers not being present. We also deployed 'face-saving' questioning techniques such as offering 'get-outs' for respondents who may have felt pressured to present themselves as caring and compassionate. Using an online mode also meant respondents could work at their own pace–particularly relevant for older respondents. The sample size is quite large for an opinion survey and this, combined with the method of sampling and weighting provided data that should yield good representation of the population, as indicated by the table on sampling error.

In common with any survey that examines claims by respondents as to how they would behave in a particular context, this research is limited by the accuracy with which responses reflect what would happen in real life. In particular, and with respect to the key findings around 'willingness to help', the authors would regard survey responses to such questions as at the upper limit of the likely 'real world' outcomes. It is also important to note the high percentage of respondents agreeing with seemingly contradictory statements indicating that some opinions may be lightly held or indicate conflicting sentiments and possibly be prone to change.

## Conclusion

Policy makers and politicians internationally face difficult choices in addressing the issue of caring for older populations at the end of life [77]. Institution-based care is expensive and seen by many as unsatisfactory, with evidence of poor quality of care in hospitals not geared up to provide palliative care. In addition, given that most of the public express a preference to end their lives at home [78], but relatively few actually do so, there remains an urgency to solve this problem.

In seeking solutions, public opinion generally, and public support for new ideas in particular, is key. In response, this work examined public opinions on possible community care models in England. The results offer initial indications that future co-operation may be possible, but suggest there is a long way to go before the English public is ready to fully embrace such ideas. In setting these results in an international context, this paper has suggested the use of collectivist-individualist measures as a possible initial benchmark indicator of a country's cultural willingness to co-operate. For those countries such as England with very high measures of individualism, there are likely to be significantly higher resources and time required to create the conditions necessary for community care to succeed.

## Supporting information

S1 File Supporting information. (DOCX)
