# Supplementary material for: Public responses to volunteer community care: Propositions for old age and end of life
Source: PLoS One. 2019 Jul 1;14(7):e0218597. doi: 10.1371/journal.pone.0218597 (PMC6602190; doi:10.1371/journal.pone.0218597)
Supplement: S1 File — (DOCX) [file pone.0218597.s001.docx]

# Supporting information

**S1 Appendix. Questionnaire.** Available from: <https://www2.uwe.ac.uk/faculties/BBS/Documents/Care_Initiative_Questionnaire.pdf>

**S2 Appendix. Report on Volunteering.**

Building and retaining groups of volunteers to help the elderly. Available from: <https://www2.uwe.ac.uk/faculties/BBS/Documents/Volunteer_Groups_for_the_elderly.pdf>

**S3 Appendix. Data including definitions.**

Available from: <https://www2.uwe.ac.uk/faculties/BBS/Documents/Care%20Initiative%20Data.xlsx>

**S4 Appendix. YouGov Privacy Policy.**

Available from: <https://yougov.co.uk/about/terms-combined/#/privacy>.

S1 Table. Sampling error associated with Table percentages.

To help judge the likely statistical reliability of the percentages in the paper the table below shows the margins of error for the base sizes and % results used in all Tables at the 95% confidence interval. Note that effective base sizes were used – these take into account the magnitude of the weighting applied to accurately reflect the population demographics of England.

| **Sample definition and unweighted base size** | **Approximate sampling tolerances applicable to percentages at or near these levels** | | |
| --- | --- | --- | --- |
|  | **10% or 90%** | **30% or 70%** | **50%** |
|  | + | + | + |
| Total adults aged 45+  n= 3590 | 1.2% | 1.8% | 1.9% |
| Have cared for an elderly person  n=2219 | 1.5% | 2.2% | 2.4% |
| Might consider joining a group  n=1150 | 2.1% | 3.2% | 3.4% |
| Would help someone not known  n=785 | 2.5% | 3.8% | 4.2% |

(Note: All figures quoted are statistically significantly different to other quoted figures at 95% CI unless signified)
